# Supplementary material for: Seizure Sources Can Be Imaged from Scalp EEG by Means of Biophysically Constrained Deep Neural Networks
Source: Adv Sci (Weinh). 2024 Oct 29;11(47):2405246. doi: 10.1002/advs.202405246 (PMC11653641; doi:10.1002/advs.202405246)
Supplement: Supplementary file 1 — Supporting Information [file ADVS-11-2405246-s001.pdf]

ADVANCED SCIENCE
Open Access

Supporting Information

for *Adv. Sci.*, DOI 10.1002/advs.202405246

Seizure Sources Can Be Imaged from Scalp EEG by Means of Biophysically Constrained Deep Neural Networks

*Rui Sun, Abbas Sohrabpour, Boney Joseph, Gregory Worrell and Bin He**

Supplementary Information

Table S1

Patient information							
Pt. #	Gender	Histopathology	Surgical Resection	Intracranial EEG	Follow-up	Outcome	Surgery age
1	F	Right temporal lobe gliosis and mesial temporal sclerosis	Right anterior temporal lobectomy and amygdalohippocampectomy	Right temporal grids and strips and depth electrodes on mesial side	1 year	ILAE-1	33
2	M	Right temporal lobe gliosis and mesial temporal gliosis	Right anterior temporal lobectomy and hippocampectomy	Bilateral depth electrodes into hippocampi	3 years	ILAE-1	26
3	F	Left temporal lobe gliosis	Left anterior temporal lobectomy and amygdalohippocampectomy	Left frontal and temporal grids and strips and depth electrodes on mesial side	3 years	ILAE-1	28
4	F	Left temporal lobe gliosis and mesial temporal sclerosis	Left anterior temporal lobectomy and amygdalohippocampectomy	Left temporal strips and mesial depth electrodes	32 months	ILAE-1	22
5	F	Left Frontal Lobe - Moderate to marked subcortical and subpial gliosis	Left anterior frontal lobe excision	N.A.	30 months	ILAE-1	22
6	F	Right temporal lobe gliosis and right amygdalohippocampectomy neuronal loss and gliosis	Right anterior temporal lobectomy and amygdalohippocampectomy	Right temporal grids and strips and depth electrodes on mesial side	1 year	ILAE-1	21
7	F	Left temporal lobe and hippocampus gliosis	Left anterior temporal lobectomy and amygdalohippocampectomy	Left frontal and temporal grids and strips and mesial depth electrodes	1 year	ILAE-2	N.A.
8	F	Right temporal lobe gliosis, mesial temporal structures and hippocampus gliosis	Right temporal lobectomy and amygdalohippocampectomy	N.A.	2 years	ILAE-2	46
9	F	Right temporal lobe gliosis and right hippocampus gliosis	Right temporal lobectomy and amygdalohippocampectomy	Right temporal grids and strips and depth electrodes on mesial side	1 year	ILAE-1	19
10	F	Left temporal lobe gliosis, left hippocampus and	Left temporal lobectomy and amygdalohippocampecomy	N.A.	32 months	ILAE-1	60

		amygdala neuronal loss and gliosis,					
11	F	Left temporal lobe gliosis, left amygdala and hippocampus neuronal loss and gliosis	Left temporal lobectomy and amygdalohippocampecomy	Right temporal grids and strips and depth electrodes on mesial side	1 year	ILAE-1	53
12	F	Right temporal lobe gliosis, right hippocampus neuronal loss with associated gliosis	Right temporal lobectomy and amygdalohippocampectomy	Right temporal grids and strips and depth electrodes on mesial side	1 year	ILAE-1	25
13	F	Right temporal lobe and right hippocampus gliosis	Extended right temporal lobectomy with amygdalohippocampectomy	Multiple right lateral temporal and sub-temporal grids and strips of electrodes	15 months	ILAE-1	38
14	M	Right temporal lobe gliosis, right hippocampus mesial temporal structures gliosis	Right temporal lobectomy and amygdalohippocampectomy	Right temporal grids and strips and depth electrodes on mesial side	1.5 years	ILAE-1	48
15	M	Right temporal low-grade astrocytoma and gliosis, right hippocampus mesial temporal structures gliosis	Right anterior temporal lobectomy and amygdalohippocampectomy	Right temporal grids and strips and depth electrodes on mesial side	1 year	ILAE-1	51
16	F	Focal Cortical Dysplasia: left parietal cortex with architectural disorganization and neuronal dysmorphism associated with marked gliosis	Left parietal focal cortical resection	Right temporal grids and strips and depth electrodes on mesial side	1 year	ILAE-1	25
17	M	Left frontal lobe gliosis	Modified left frontal lobectomy	Left frontal strips	19 months	ILAE-1	21
18	M	Right temporal lobe vascular malformation, gray and white matter focal gliosis	Right temporal lesion resection	N.A.	19 months	ILAE-1	49
19	F	First and second surgery: right frontal lobe gliosis	Right frontal cortical resection	Right frontal, orbital frontal, and bilateral interhemispheric grid/stripes/depth electrodes	14 months	ILAE-1	15
20	F	Left temporal lobe and hippocampus gliosis	Left anterior temporal lobectomy and amygdalohippocampectomy	Left parietal, left temporal mesial, and lateral neocortical region depth,	16 months	ILAE-1	23

				subdural grid, and strip electrodes			
21	M	Right frontal lobe gliosis	Right mesial frontal orbital lobe excision	Bilateral temporal and frontal depth electrodes	15 months	ILAE-1	25
22	M	Right frontotemporal lobe arteriovenous malformation and gliosis, cortical microinfarctions, embolic material in vessels, and white matter gliosis.	Right frontotemporal lobe resection	Right temporoparietal and right parietal grid, right temporal depth electrodes	2 years	ILAE-5	25
23	M	Right temporal lobe and amygdala gliosis	Right temporal lobectomy and amygdalohippocampectomy	Bitemporal depth electrodes	9 years	ILAE-5	39
24	F	Right temporal lobe gliosis; amygdala and hippocampus neuronal loss and gliosis	Right temporal lobectomy and amygdalohippocampectomy	Right temporal strips and mesial temporal depth electrodes	19 months	ILAE-3	50
25	M	Right medial temporal lobe gliosis; right hippocampus neuronal loss and gliosis	Right anterior temporal lobectomyand amygdalohippocampectomy	Right temporal strips and mesial temporal depth electrodes	18 months	ILAE-4	29
26	F	Left temporal gliosis, leptomeningeal mixed inflammation; amygdalohippocampus having electrode implantation site identified	Left anterior temporal lobectomy and amygdalohippocampectomy	Left temporal grid and strips on the inferior temporal side and left frontal, depth electrodes on temporal and frontal	12 months	ILAE-4	22
27	F	First surgery: right temporo-occipital gliosis; Second surgery: right temporo-occipital gliosis, leptomeningeal and superficial cortical inflammation	Focal temporo-occipital cortical resection	Right fronto-temporal grids and strips and depth electrodes close to amygdala and hippocampus, and later right posterotemporo-parietal grids and strips	12 months	ILAE-4	22
28	M	right amygdalohippocampectomy gliosis and minimal glial atypica; right temporal lobe gliosis	Right temporal lobectomy and amygdalohippocampectomy	Right temporal strips	13 months	ILAE-4	29
29	F	left temporal lobe gliosis; left hippocampus with mesial temporal sclerosis	Left temporal lobectomy and amygdalohippocampectomy	left temporal strips and mesial depth electrodes	15 months	ILAE-4	58

30	F	Left and right temporal lobe gliosis	Left anterior temporal lobectomy and amygdalohippocampectomy	Left temporal strips and electrodes	12 months	ILAE-3	N.A.
31	M	N.A.	Lateral temporal extending into parietal cortex resection	Left subdural grids, strips, and depth electrodes	14 months	ILAE-5	34
32	M	Right Frontal Lobe - Focal cortical dysplasia	Posterior superior margin of the sylvian fissure resection	Right subdural grids, strips, and depth electrodes	15 months	ILAE-5	37
33	M	Left posterior temporo-occipital gliosis; left lateral temporal lobe matter gliosis; left hippocampus severe pyramidal neuronal loss and gliosis.	Left anterior temporal lobectomy, amygdalohippocampectomy, and left posterior temporo-occipital corticectomy	Left temporal subdural grids, strip electrodes, and left depth electrodes	15 months	ILAE-3	47

Figures

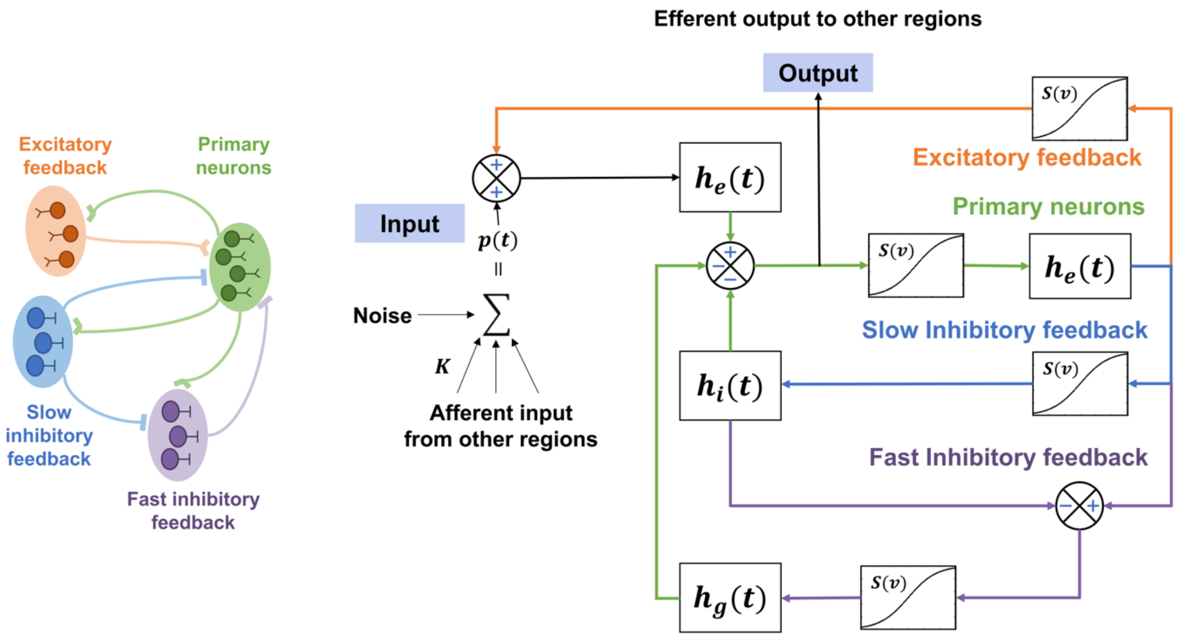

Figure S1. *A single-element modified Jansen-Rit model simulates four neuronal sub-populations: the primary neurons (i.e., pyramidal cells), as well as excitatory, slow inhibitory and fast inhibitory interneurons. The influence from other neural ensembles is modeled as an excitatory input p(t) that represents the average pulse density, i.e., average firing rate, of afferent action potentials. The behavior of each subpopulation is modeled using linear functions* $h(t)$ *that describe how presynaptic information, from excitatory and inhibitory populations, is transformed into postsynaptic information, and nonlinear function S(v) that models the change from average membrane potential of the subpopulation to average pulse density.*

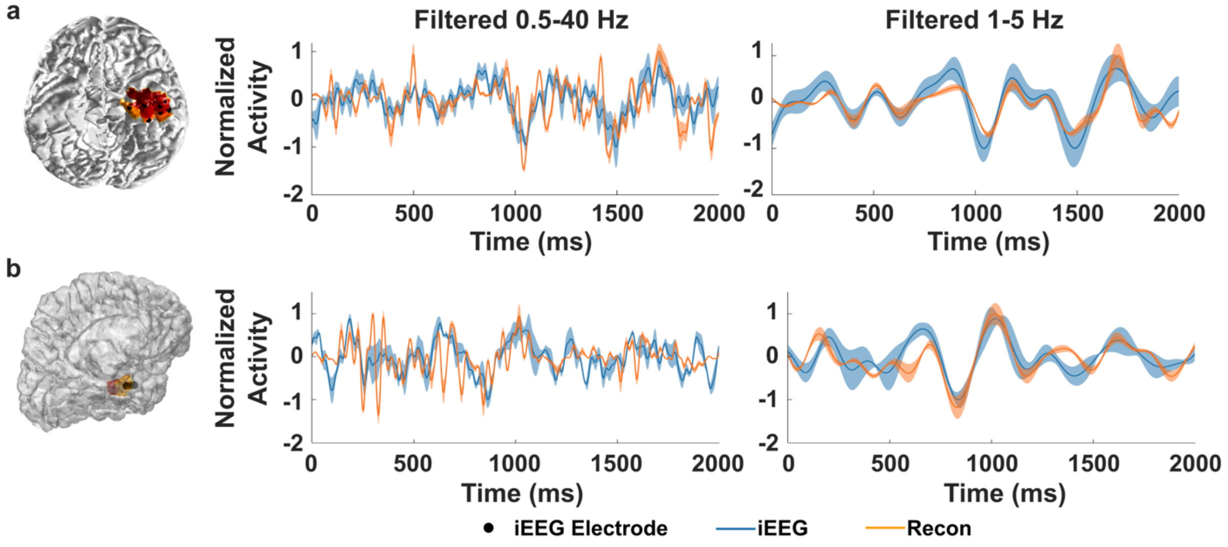

***Figure S2**. Comparing estimated time-courses from EEG to intracranial EEG recordings in 2 patients. The reconstructed time-courses near the SOZ electrodes from DeepSIF were averaged and compared to the averaged iEEG traces. The major ictal frequency for these patients is around 3 Hz. **a,** The linear correlation between the iEEG and reconstruction is 0.5 for wide-band signal, and 0.84 for narrow-band signal filter around the major frequency. **b,** The linear correlation between the iEEG and reconstruction is 0.4 for wide-band signal, and 0.77 for narrow-band signal filter around the major frequency.*

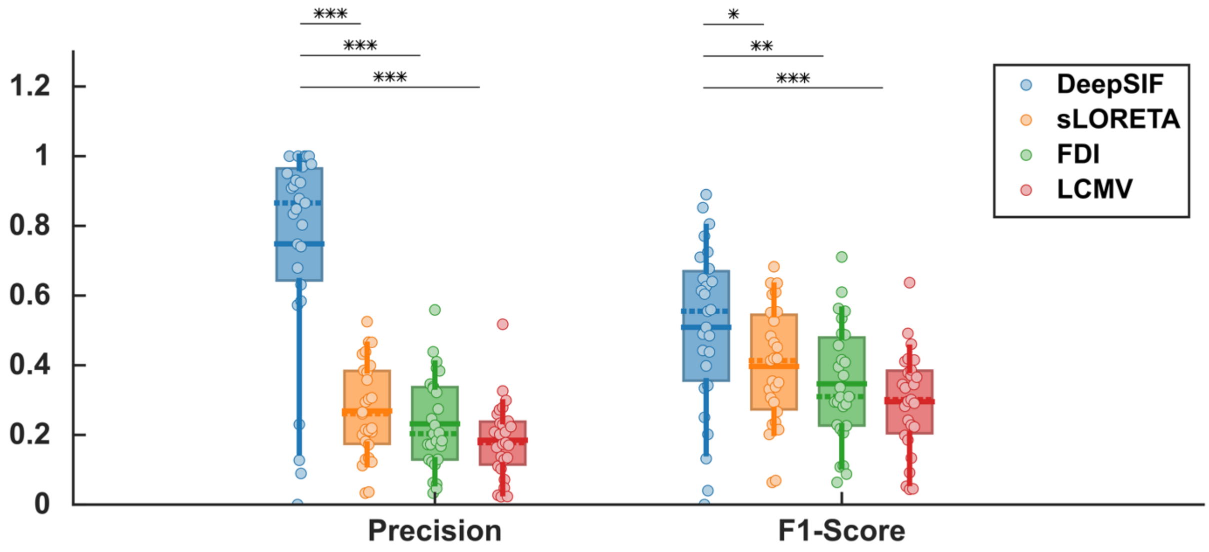

Figure S3. *Precision and F1-Score for different ictal source imaging methods. n = 27. Precision: 0.75 ± 0.30 [D], 0.27 ± 0.14 [S], 0.23 ± 0.13 [F], 0.18 ± 0.11 [L]; F1-Score: 0.51 ± 0.24 [D], 0.40 ± 0.17 [S], 0.35 ± 0.17 [F], 0.30 ± 0.14 [L], where [D], [S], [F], and [L] represent DeepSIF, sLORETA, FDI, and LCMV, respectively. Paired one-sided Wilcoxon signed rank test was used with statistical significance cutoffs of (*P<0.05, **P<0.01, ***P<0.001).*

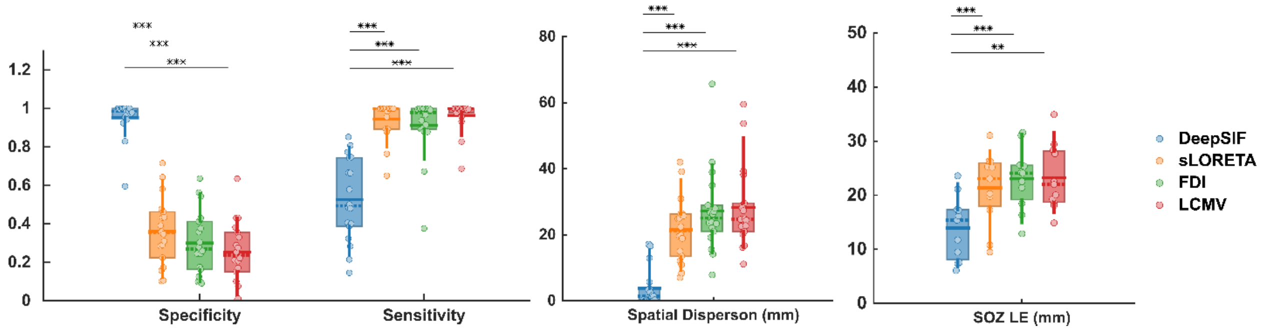

Figure S4. *Ictal source imaging results for different ictal imaging methods in the seizure free group. Spatial specificity. n = 16, 0.95 ± 0.10 [D], 0.36 ± 0.18 [S], 0.30 ± 0.17 [F], 0.25 ± 0.16 [L]; Spatial sensitivity: n = 16, 0.52 ± 0.21 [D], 0.95 ± 0.10 [S], 0.91 ± 0.16 [F], 0.96 ± 0.08 [L]; Spatial dispersion (mm): n = 16, 3.83 ± 5.68 [D], 21.57 ± 9.73 [S], 27.20 ± 12.71 [F], 28.29 ± 12.47 [L]; SOZ localization error (LE) (mm): n = 6, 13.92 ± 5.87 [D], 21.34 ± 6.69 [S], 23.05 ± 5.69 [F], 23.24 ± 6.06 [L], where [D], [S], [F], and [L]represent DeepSIF, sLORETA, FDI, and LCMV, respectively. Paired one-sided Wilcoxon signed rank test was used with statistical significance cutoffs of (*P<0.05, **P<0.01, ***P<0.001).*

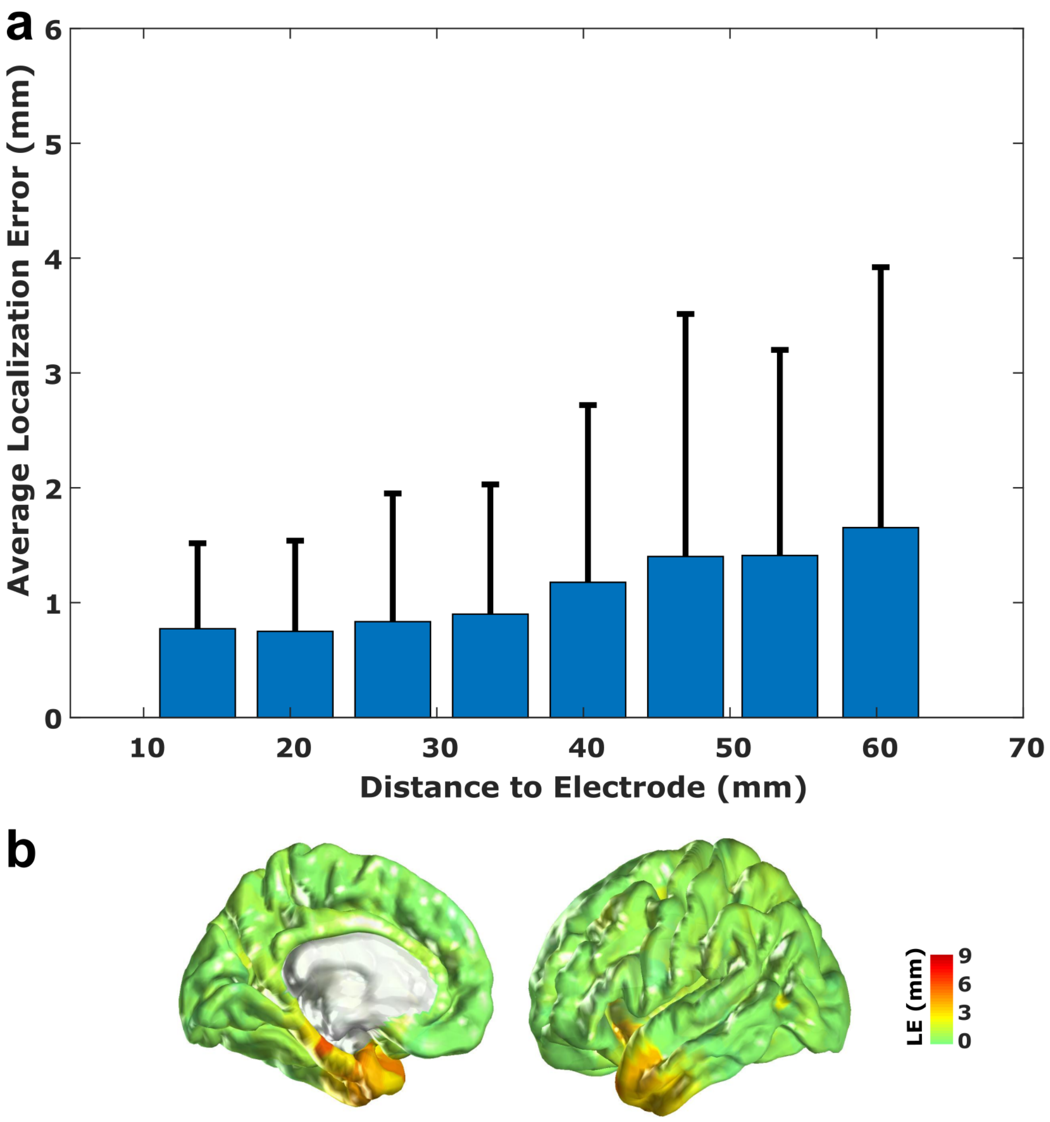

Figure S5. Simulation results on localization errors across the cortex and along different source depth, with temporal dynamics varying in time (n = 23,856) for all SNR levels (5-20 dB). (a) The plot shows the average localization error for all sources within a particular depth. The error bar shows the standard deviation. (b) The mean localization error for all source locations displayed on the cortex.

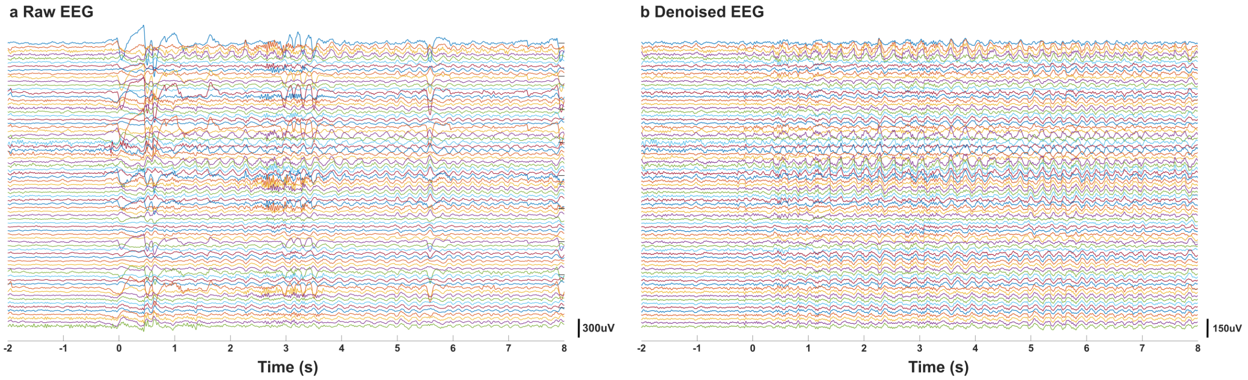

Figure S6. An example of seizure signal preprocessing. One example of 2 seconds before and 8 seconds after the seizure onset is shown for a, raw EEG signal and b, ICA cleaned EEG signal.

Supplementary Video

Ictal source imaging results on a patient of left temporal lobe epilepsy (Patient #3, ILAE-1) are shown in this video. In a 5-second signal from seizure onset, the topological map (top left) and the DeepSIF source imaging result (right) are shown. A threshold calculated using the Otsu's thresholding technique is used on the imaging result. The surface EEG channel with the maximum energy is plotted. The resection region is shown in green and the iEEG SOZ electrodes are shown in red as a reference. The reconstructed noninvasive DeepSIF source imaging results are in full concordance with the clinical findings determined from invasive recordings and surgical resection outcome.
